# Editorial: Post COVID-19 physical performance and functional capacity

**DOI:** 10.3389/fmed.2022.920645

**Published:** 2022-07-29

**Authors:** Klara Komici, Leonardo Bencivenga, Giuseppe Rengo

**Affiliations:** ^1^Department of Medicine and Health Sciences, University of Molise, Campobasso, Italy; ^2^Department of Advanced Biomedical Sciences, University of Naples Federico II, Naples, Italy; ^3^Gérontopôle de Toulouse, Institut du Vieillissement, CHU de Toulouse, Toulouse, France; ^4^Department of Translational Medical Sciences, University of Naples “Federico II”, Naples, Italy; ^5^Istituti Clinici Scientifici Maugeri SpA Societa Benefit (ICS Maugeri SpA SB), Telese Terme, Italy

**Keywords:** SARS-CoV-2, COVID-19, physical performance, functional capacity, post-COVID-19

The SARS-CoV-2 infection can range from being asymptomatic to presenting with a wide spectrum of clinical manifestations including symptoms such as fever, fatigue, and myalgia, as well as respiratory, cardiovascular, neurological, and gastrointestinal problems (1, 2). Furthermore, the pandemic has impacted individuals' health through indirect effects, such as in-person healthcare visits, and the diagnosis, treatment, and monitoring of other acute and chronic conditions (3). In particular, healthcare assessments have decreased by about a third, with greater reductions among people with less severe illnesses (4).

Beyond the severe clinical manifestations of acute Coronavirus Disease 2019 (COVID-19), about 80% of patients develop one or more long-term symptoms (5). Fatigue, dyspnea, headache, attention alteration, anxiety, mood disorders, and significant decline in quality of life are commonly reported symptoms associated with post-acute sequelae of SARS-CoV-2 infection (5, 6). Several studies have described a reduction of physical performance in COVID-19 survivors, as well as a worsening of functional ability, leading to a loss of independence following the acute phase of SARS-CoV-2 infection (7, 8). Particularly in the case of prolonged hospitalizations, especially for critical illness, impaired physical performance is common and prior functional limitations are exacerbated. In the specific context of post-COVID-19, these deleterious effects may be further attributed to prolonged immobility and physical deconditioning, in addition to the pathophysiological mechanisms directly related to SARS-CoV-2 systemic inflammation and multiorgan damage.

Based on these important considerations, the present Research Topic aimed to collect the most updated evidence on the impact of the pandemic on functional capacity, physical performance, and post-COVID-19 recovery, with a specific focus on the multidimensional performance of older adults.

Fujita et al. reviewed data on COVID-19 patients requiring hospitalization between April 2020 and March 2021. Almost two out of five hospitalized patients were over 80 years old and ~40% of patients suffered from impairment of Activities of Daily Living (ADL) on admission. Older patients required significantly longer periods of hospitalization compared to those under 80 years of age. Moreover, immobilization, isolation, nutritional impairment, and delirium were the most frequent complications during hospitalization in these older patients, suggesting a greater burden on healthcare providers and raising the question of an increased need for resource allocation in terms of healthcare personnel. As expected, the in-hospital mortality rate was higher among older patients, and a greater level of dependence was significantly associated with increased mortality. Notably, limitation in self-care and functional abilities, as assessed through ADL, was associated with higher dependency and negatively influenced the quality of life of both patients and caregivers. Similarly, another study reported an increase in in-hospital mortality in COVID-19 patients with impairment in more than two ADL functions (9). Dependency, disability, and frailty are key elements associated with poorer prognosis among geriatric patients in different clinical settings (10, 11). Moreover, dependency and disability are mainly associated with advanced-stage organ failure and dementia, which was the most frequent concomitant disease in the study of Fujita et al.

Physical activity is well known to contribute to healthy aging and has been shown to have beneficial effects on functional status, comorbidities, and frailty status. Cerasola et al. discussed the importance of physical exercise in the context of the COVID-19 pandemic, particularly the benefits of exercise training on the pandemic burden and long-COVID-19 syndrome. Physically active patients seem not to experience a reduction in physical performance secondary to SARS-CoV-2 infection and generally the presentation is limited to mild or asymptomatic disease (12, 13). While radical changes in lifestyle during the pandemic were associated with inevitable detrimental effects on mood and mental health, regular physical activity helped to improve symptoms of depression and reduce anxiety (14). The negative effects of social isolation due to COVID-19 may result in a more sedentary lifestyle, which negatively influences mental health and worsens the management of comorbidities in older people. Indeed, social distancing, quarantine, and home isolation constitute obstacles to physical exercise and training and impair motivation. Therefore, the research group led by Chang et al. highlights the role of home-based exercise training using an online platform for improving the functional fitness of community-dwelling adults. In this single-blinded study, the intervention consisted of two training sessions (75–90 mins each) per week, comprised of flexibility, balance, and cardio-vascular fitness exercises. The results suggest a beneficial effect of this program on improving lower limb flexibility, muscle strength, and cardiorespiratory fitness in this sample of community-dwelling older adults.

Bordas-Martines et al. report that in survivors of severe COVID-19 infections, an early intervention of physical therapy training consisting of 10–15 mins of pulmonary and muscolo-skeletal rehabilitation administered 5–7 days per week was not associated with adverse effects nor with a worsening of clinical conditions. Importantly, no infections with COVID-19 were registered among physiotherapists during the study period. In addition, both the length of hospitalization and the time to achieve a sitting position were shorter in the early physical therapy group compared to controls. Nevertheless, dyspnea, walking capacity, self-reported quality of life, and persistent chest-X-ray infiltrates did not significantly change between early or delayed rehabilitation. Of note, in post-acute COVID-19 patients, rehabilitation was associated with preserved ADL and increased grip strength (15).

Pasini et al. observed that patients with a new onset of fatigue, muscle weakness, and/or dyspnea, who were asymptomatic prior to SARS-CoV-2 infection, show post healing higher serum levels of ferritin, inflammatory markers such as C-reactive Protein, and LDH, lower albumin, altered coagulation and increased D-Dimer. These findings raise the question of the role of systemic inflammation, activation of cellular autophagy, apoptosis, and necrosis in the clinical presentation of post-acute sequelae of SARS-CoV-2 infection (PASC). The release of inflammatory factors may in turn promote a state of chronic inflammation with metabolic implications and contribute to the clinical presentation, leading to symptoms such as fatigue, myalgia, or exhaustion. The consequences of these alternations are still unknown but lifestyle interventions, such as the correction of nutritional status (16), may be necessary to restore the metabolic and immune-inflammatory alterations.

This Research Topic explores the intricate links between SARS-CoV-2 infection and impairment in functional capacity and physical performance. Post-COVID-19 syndrome, characterized by further reduction of functional capacity or by the new onset of dependency and exercise intolerance, may be related to the presence of comorbidities, immunosenescence, and inflammation. Active physical rehabilitation and the implementation of structured interventional programs may constitute key factors in preventing dependency and improving the management of post-COVID-19 symptoms in older patients.

## Author contributions

KK wrote the manuscript. LB and GR revised the manuscript. All authors contributed to the article and approved the submitted version.

## Conflict of interest

The authors declare that the research was conducted in the absence of any commercial or financial relationships that could be construed as a potential conflict of interest.

## Publisher's note

All claims expressed in this article are solely those of the authors and do not necessarily represent those of their affiliated organizations, or those of the publisher, the editors and the reviewers. Any product that may be evaluated in this article, or claim that may be made by its manufacturer, is not guaranteed or endorsed by the publisher.
